# Risk factors for dysphagia after anterior cervical spine surgery: A meta-analysis

**DOI:** 10.1097/MD.0000000000006267

**Published:** 2017-03-10

**Authors:** Feng-Yu Liu, Da-Long Yang, Wen-Zheng Huang, Li-Shuang Huo, Lei Ma, Hui Wang, Si-Dong Yang, Wen-Yuan Ding

**Affiliations:** a Department of Spinal Surgery, The Third Hospital of Hebei Medical University, Shijiazhuang; b Department of Orthopaedics, General Hospital of Fengfeng Corporation of Jizhong Energy Group, Handan; c Department of Endocrinology, The Second Hospital of Hebei Medical University; d Hebei Provincial Key Laboratory of Orthopedic Biomechanics, Shijiazhuang, China.

**Keywords:** cervical spinal surgery, dysphagia, meta-analysis, risk factors

## Abstract

**Background:**

Dysphagia is a well-known complication following anterior cervical spine surgery. Although risk factors for dysphagia have been reported in the literature, they still remain controversial. This study aims to investigate the risk factors associated with dysphagia following anterior cervical spinal surgery.

**Methods:**

PubMed, EMBASE, and The Cochrane Library were searched up to June 2016 for studies examining dysphagia following anterior cervical spinal surgery. Risk factors associated with dysphagia were extracted. Odds ratios (ORs) and 95% confidence intervals (CIs) were calculated for outcomes. Data analysis was conducted with RevMan 5.3 and STATA 12.0.

**Results:**

The final analysis includes a total of 18 distinct studies. The pooled analysis reveals that there are significant differences in female gender (OR = 2.30, 95% CI: 1.76–2.99, *P* < 0.001), the use of anterior cervical plate (OR = 1.66, 95% CI: 1.05–2.62, *P* = 0.03), more than 1 surgical level (OR = 2.07, 95% CI: 1.62–2.66, *P* < 0.001), the upper surgical level at C3/4 (OR = 3.08, 95% CI: 1.44–6.55, *P* = 0.004), and the use of bone morphogenetic protein-2 (rhBMP-2) (OR = 5.52, 95% CI: 2.16–14.10, *P* < 0.001). However, no significant difference is found in revision surgery (OR = 1.67, 95% CI: 0.60–4.68, *P* = 0.33), the type of fusion (OR = 1.02, 95% CI: 0.62–1.67, *P* = 0.95), and cervical disc arthroplasty (OR = 1.37, 95% CI: 0.75–2.51, *P* = 0.30).

**Conclusion:**

Female gender, the use of anterior cervical plate, more than 1 surgical level, the upper surgical level at C3/4, and the use of rhBMP-2 are the risk factors for dysphagia following anterior cervical spinal surgery. However, revision surgery, the type of fusion, and cervical disc arthroplasty are unassociated with dysphagia. Considering the limited number of studies, this conclusion should be interpreted cautiously, and larger scale studies are required.

## 1 Introduction

Anterior cervical spine surgery is commonly performed for the treatment of cervical spine pathologies, including trauma and degenerative spinal diseases.^[1]^ The anterior approach is safe, effective, and has low rate of morbidity and mortality. However, a number of complications associated with the anterior approach have been described.^[2]^ Dysphagia is reported as one of the most common early complaints after anterior cervical spine surgery. The incidence of dysphagia varies in the literature from 1% to 79%.^[3]^

The pathophysiology of dysphagia after anterior cervical spine surgery has not been well understood. A large number of risk factors, such as multilevel surgery, revision surgery, gender, the use of hardware, and the use of bone morphogenetic protein-2 (rhBMP-2), are associated with an increase in postoperative dysphagia incidence.^[3,4]^

Several studies, based on an analysis in small sample size, have reported the risk factors associated with dysphagia. Meta-analysis, as a great statistical method, can be used to combine the results from multiple studies to improve estimates of the magnitude of an effect, strengthen statistical power, and solve uncertainty across conflicting reports. Thus, a meta-analysis was carried out in an effort to assess the risk factors associated with dysphagia following anterior cervical spinal surgery.

## 2 Materials and methods

### 2.1 Ethics statement

No effort is needed to seek consent from patients, because all the data collected and analyzed in this study are anonymous and do no potentially harm the patients. Ethical approval is unnecessary for the paper.^[30]^

### 2.2 Search strategy

PubMed, EMBASE, and The Cochrane Library were extensively searched; and it was completed on June 1, 2016. There was no restriction on the year of publication. The language was restricted to English, and only published articles were included. The searching string was applied as (dysphagia OR swallowing disorders OR swallowing dysfunction) AND risk factors AND anterior AND cervical. References cited in the relevant literatures were also reviewed.

### 2.3 Selection criteria

Studies were included based on the following criteria: randomized or nonrandomized controlled studies; patients with cervical spondylotic myelopathy, cervical canal stenosis, or ossification of posterior longitudinal ligament; patients undergoing anterior cervical spinal surgery including anterior cervical decompression and fusion (ACDF), anterior cervical corpectomy and fusion (ACCF), or cervical disc arthroplasty; measured outcomes of risk factors for dysphagia; follow-up time of at least 3 months. The exclusion criteria were as follows: case reports, reviews, or letters; repeatedly published data; and unreported outcomes of interest. The potentially qualified studies were selected independently by 2 reviewers (FYL and DLY) according to the inclusion and exclusion criteria. Any discrepancy was addressed through discussion, and consensus was reached.

### 2.4 Data extraction

Data were extracted individually by 2 authors (FYL and WZH). By discussion or by involving a third author (WYD), disagreements were addressed. The general features cover first author, study design, follow-up time, year of publication, country, and sample size. The results include gender, the use of hardware, revision surgery, the type of fusion (ACCF or ACDF), the use of rhBMP-2, surgical level, and cervical disc arthroplasty.

### 2.5 Quality assessment

The Newcastle Ottawa Quality Assessment Scale (NOQAS) was utilized to evaluate the quality of each study, since most studies included are nonrandomized controlled studies. To allocate a maximum of 9 points for the quality of selection, exposure, comparability, and results for study participants, this scale for nonrandomized case-controlled studies and cohort studies was applied.^[29]^

### 2.6 Statistical analysis

This study only mentioned dichotomous outcomes, so odds ratios (ORs) and 95% confidence intervals (CIs) were calculated for outcomes. A *P* value <0.05 was counted as statistically significant. A random-effects or fixed-effects model was applied based on the heterogeneity of the studies included. Heterogeneity was analyzed with *I*^2^ test, in which *I*^2^ > 50% implies heterogeneity.^[29]^ All statistical analyses were conducted by using RevMan 5.3 (The Cochrane Collaboration, Oxford, UK) and STATA 12.0 (Stata Corporation, College Station, TX). Sensitivity analysis was conducted to examine the influence of excluding each study. Potential publication bias was assessed using funnel plot, Egger linear regression test, Begg rank correlation test, and trim and fill method.

## 3 Results

### 3.1 Search results

**Figure 1:**
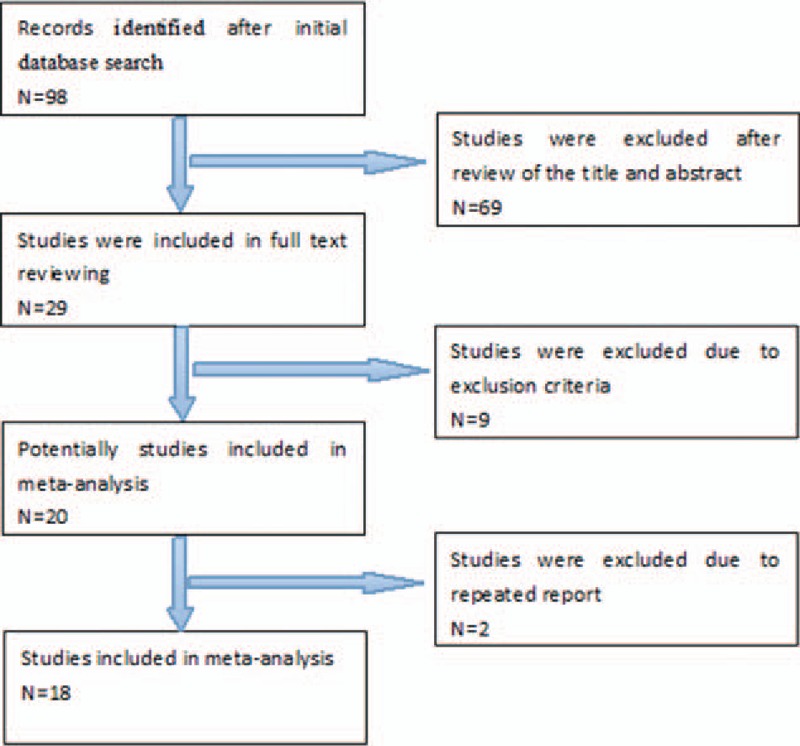
Flow diagram of study selection.

The initial database search identified a total of 98 records. After the titles and abstracts were reviewed, 69 of them were eliminated. A full-text review was evaluated in the 29 records maintained, and 9 of them were excluded because no outcome of interest is provided. Another 2 were eliminated because their data from the American Nationwide Inpatient Sample database might be repeated with other studies. Finally, 18 articles meeting the inclusion criteria were included in the present meta-analysis. Figure 1 shows the selection process.

### 3.2 Baseline characteristics

Eighteen studies published from 2002 to 2014 were included in this meta-analysis. Their size ranges from 17 to 463 patients (a total of 2891). Table 1 presents the characteristics of those included studies.

**Table 1:**
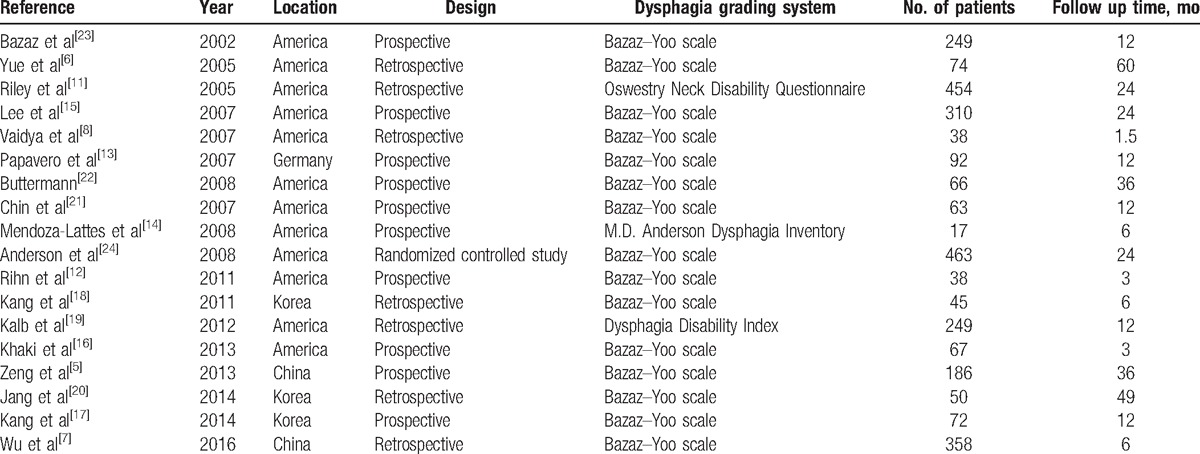
Characteristics of included studies.

### 3.3 Quality assessment

Only 1 is a randomized controlled study, and all the other 17 are nonrandomized controlled studies, including 7 retrospective and 10 prospective studies. To evaluate the quality of each study, the NOQAS was utilized. In those studies, 12 of them scores 8 points and 6 scores 7 points. Hence, the quality of each study is relatively high (Table 2).

**Table 2:**
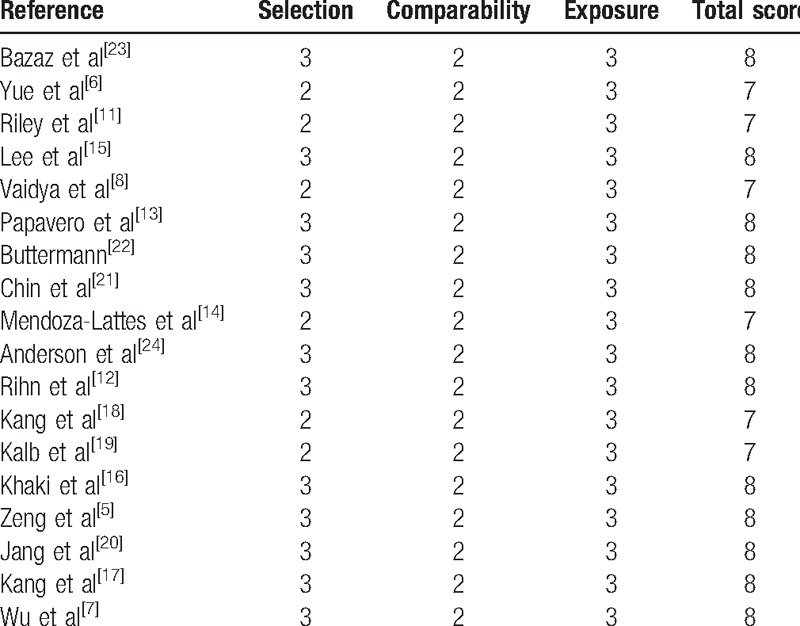
The quality assessment according to the Newcastle Ottawa Quality Assessment Scale of each study.

### 3.4 Assessment of risk factors for dysphagia

**Figure 2:**
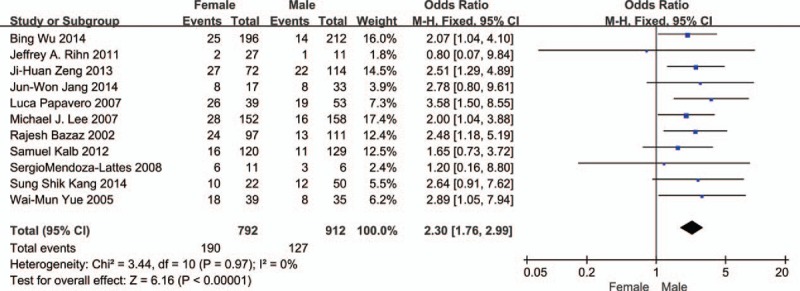
The odds ratio estimate for gender.

Eleven studies reported the relationship between gender and dysphagia (Fig. 2). The test for heterogeneity was insignificant, and the studies had low heterogeneity (*P* for heterogeneity = 0.97; *I*^2^ = 0%). The fixed-effect model was used. The aggregated results of the 11 studies suggested that female gender was a risk factor for dysphagia following anterior cervical spinal surgery (OR = 2.30, 95% CI: 1.76–2.99, *P* < 0.001).

Two studies presented the relationship between revision surgery and dysphagia. The test for heterogeneity was significant, and the studies had high heterogeneity (*P* for heterogeneity = 0.10; *I*^2^ = 64%). The random-effect model was used. The aggregated results of the 2 studies indicated that revision surgery was unrelated to dysphagia (OR = 1.67, 95% CI: 0.60–4.68, *P* = 0.33).

**Figure 3:**
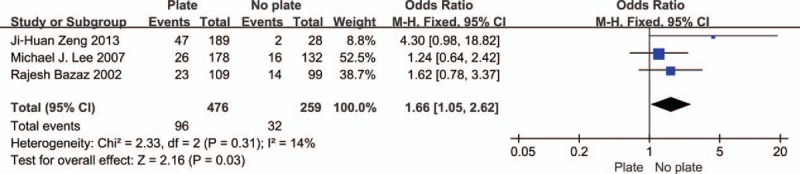
The odds ratio estimate for use of anterior cervical plate.

Three studies revealed the relationship between the use of anterior cervical plate and dysphagia (Fig. 3). The test for heterogeneity was insignificant, and the studies had low heterogeneity (*P* for heterogeneity = 0.31; *I*^2^ = 14%). The fixed-effect model was used. The aggregated results of the 3 studies suggested that use of anterior cervical plate was a risk factor for dysphagia following anterior cervical spinal surgery (OR = 1.66, 95% CI: 1.05–2.62, *P* = 0.03).

Two studies reported the relationship between the type of fusion (ACCF and ACDF) and dysphagia. The test for heterogeneity was insignificant, and the studies had low heterogeneity (*P* for heterogeneity = 0.21; *I*^2^ = 38%). The fixed-effect model was used. The aggregated results of the 2 studies manifested that type of fusion was not associated with dysphagia (OR = 1.02, 95% CI: 0.62–1.67, *P* = 0.95).

**Figure 4:**
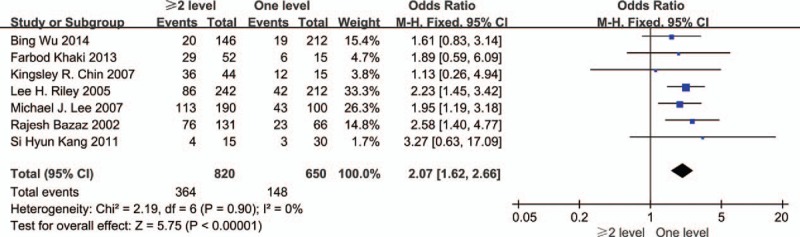
The odds ratio estimate for multiple surgical levels.

Seven studies showed the relationship between multiple surgical levels and dysphagia (Fig. 4). The test for heterogeneity was insignificant, and the studies had low heterogeneity (*P* for heterogeneity = 0.90; *I*^2^ = 0%). The fixed-effect model was used. The aggregated results of the 7 studies revealed that more than 1 surgical level was a risk factor for dysphagia following anterior cervical spinal surgery (OR = 2.07, 95% CI: 1.62–2.66, *P* < 0.001).

**Figure 5:**
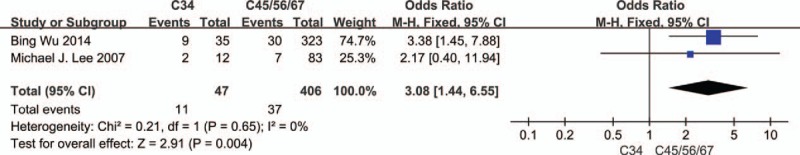
The odds ratio estimate for upper cervical spine surgery.

Two studies reported the relationship between upper cervical spine surgery and dysphagia (Fig. 5). The test for heterogeneity was insignificant, and the studies had low heterogeneity (*P* for heterogeneity = 0.65; *I*^2^ = 0%). The fixed-effect model was used. The aggregated results of the 2 studies suggested that the upper surgical level at C3/4 was a risk factor for dysphagia following anterior cervical spinal surgery (OR = 3.08, 95% CI: 1.44–6.55, *P* = 0.004).

**Figure 6:**
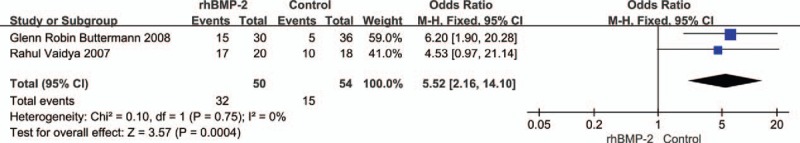
The odds ratio estimate for use of bone morphogenetic protein-2.

Two studies reported the relationship between the use of rhBMP-2 and dysphagia (Fig. 6). The test for heterogeneity was insignificant, and the studies had low heterogeneity (*P* for heterogeneity = 0.75; *I*^2^ = 0%). The fixed-effect model was used. The aggregated results of the 2 studies suggested that the use of rhBMP-2 was a risk factor for dysphagia following anterior cervical spinal surgery (OR = 5.52, 95% CI: 2.16–14.10, *P* < 0.001).

Two studies reported the relationship between cervical disc arthroplasty and dysphagia. The test for heterogeneity was insignificant, and the studies had low heterogeneity (*P* for heterogeneity = 0.34; *I*^2^ = 0%). The fixed-effect model was used. The aggregated results of the 2 studies suggested that cervical disc arthroplasty had no association with dysphagia (OR = 1.37, 95% CI: 0.75–2.51, *P* = 0.30).

### 3.5 Sensitivity analysis

To confirm the stability of the meta-analysis, a sensitivity analysis was performed by sequentially omitting individual eligible studies. The pooled prevalence was not materially changed after any single study was excluded, which indicates the stability of the results.

### 3.6 Publication bias

Assessment of publication bias for all included studies was conducted by the funnel plot on visual inspection, Egger linear regression test, Begg rank correlation test, and trim and fill method.^[30]^ For the 7 studies reporting the relationship between multiple surgical levels and dysphagia, the funnel plot shows no publication bias in multiple surgical levels (Begg, *P* = 0.764; Egger, *P* = 0.894). For 11 studies reporting the relationship between gender and dysphagia, the funnel plot demonstrates a slight asymmetry in gender (Begg, *P* = 0.161; Egger, *P* = 0.014). But the trim and fill method indicates that no study might have been missed, which suggests a reliable analysis.

## 4 Discussion

Dysphagia contributes to higher self-reported disability and lower physical health status. Persistent and severe dysphagia may lead to some catastrophic consequences such as difficulty in eating or drinking and pneumonia.^[5]^ However, the pathophysiology and risk factors of postoperative dysphagia are not fully understood. Though a large number of risk factors for dysphagia after anterior cervical spine surgery have been reported, yet almost all of them are controversial.^[6]^ Thus, a meta-analysis was performed. The pooled results from this meta-analysis suggest that female gender, the use of anterior cervical plate, multiple surgical levels, upper cervical spine surgery, and the use of rhBMP-2 are the risk factors for dysphagia following anterior cervical spinal surgery. However, revision surgery, the type of fusion, and cervical disc arthroplasty are not associated with dysphagia.

Female gender, compared with male, demonstrates a significant association with dysphagia. The reason still remains unknown but the following viewpoints may explain it. First, female gender has overall small anatomic structure, and the strength of the muscle and soft tissue is weak.^[5]^ Second, female patients, compared with male patients, have a higher sensitivity to painful stimuli.^[6]^ Third, male patients may experience more difficulty, which gives them larger average neck size and retraction needs.^[23]^

Although all of the 3 included studies show that the difference is not statistically significant, the aggregated results suggest that the use of anterior cervical plate is a risk factor for dysphagia. Hardware complication is a known etiology of postoperative dysphagia.^[15]^ According to Fogel and McDonnell,^[25]^ after the cervical instrumentation is removed, the dysphagia will be improved. Anterior cervical plate occupies a certain clearance anterior to cervical vertebra and may oppress the posterior wall of esophagus. It may disturb the normal esophageal peristalsis and cause esophageal ischemic injury that may result in dysphagia.^[5]^

More than 1 surgical level reveals significant association with dysphagia. According to Frempong-Boadu et al,^[26]^ dysphagia can be caused by soft tissue swelling. As surgical levels rise, the injury and traction to the soft tissue increase, which makes the soft tissue swelling escalate.^[11]^ Therefore, the incidence of postoperative dysphagia rises.

The upper surgical level at C3/4 presents significant association with dysphagia. On one hand, when the surgical site locates the upper cervical spine, the chance of superior laryngeal nerve damage increases and so does the incidence of postoperative dysphagia.^[7]^ On the other hand, anatomical features in upper cervical spine may also illustrate it. As the retropharyngeal space of upper cervical spine is bigger than inferior cervical spine, the soft tissue swelling will be more severe, which makes the postoperative dysphagia aggravate.^[15]^

As the use of rhBMP-2 can contribute to bone regeneration, it is used in spinal fusion operation.^[28]^ However, there are several problems with its use, including the potential for ectopic bone formation and dysphagia following anterior cervical spinal surgery. The pooled results from this meta-analysis suggest that the use of rhBMP-2 is a risk factor for dysphagia. It indicates that the use of rhBMP-2 may cause severe swelling in prevertebral soft tissues, and this effect is likely due to an early local inflammatory response to rhBMP-2.^[10]^

Although the other 3 factors are controversial, the aggregated results demonstrate that revision surgery, the type of fusion, and cervical disc arthroplasty are unassociated with dysphagia. Since the relevant literature is less, the reliability of the final results may be reduced. However, for each main outcome, the test for heterogeneity is not significant, and the studies have low heterogeneity. Sensitivity analysis and assessment of publication bias suggest the stability of the results. Thus, the aggregated results of this article are relatively reliable.

To reduce the incidence of dysphagia, some measures need to be taken based on risk factors associated with dysphagia following anterior cervical spinal surgery. First, patients, especially female ones, can perform tracheal exercises before the surgical procedure.^[9]^ Second, surgeons can shorten operative time and avoid the use of rhBMP-2.^[24]^ Finally, surgeons can use smaller and smoother cervical plates and steroid before wound closure, especially for multiple surgical levels.^[27]^

This study has its limitations. First, the dysphagia grading systems in the included studies are not exactly the same. Second, severe preoperative neck pain, older age, blood loss, and operative time are not evaluated, because related studies are few and the pooled results are unavailable. Third, the results might be impacted by the follow-up time varying between the studies. Finally, there was only 1 randomized controlled study, and all the other 17 were nonrandomized controlled studies.

## 5 Conclusion

Female gender, the use of anterior cervical plate, more than 1 surgical level, upper cervical spine surgery, and the use of rhBMP-2 are risk factors for dysphagia following anterior cervical spinal surgery. However, revision surgery, the type of fusion, and cervical disc arthroplasty are unrelated to dysphagia. Given the limitations noted above, a well designed and multicenter study needs to be conducted in the future.
